# Comprehensive analysis of a novel cuproptosis-related lncRNA signature associated with prognosis and tumor matrix features to predict immunotherapy in soft tissue carcinoma

**DOI:** 10.3389/fgene.2022.1063057

**Published:** 2022-12-07

**Authors:** Binfeng Liu, Ke Pang, Chengyao Feng, Zhongyue Liu, Chenbei Li, Haixia Zhang, Ping Liu, Zhihong Li, Shasha He, Chao Tu

**Affiliations:** ^1^ Department of Orthopaedics, The Second Xiangya Hospital of Central South University, Changsha, Hunan, China; ^2^ Hunan Key Laboratory of Tumor Models and Individualized Medicine, The Second Xiangya Hospital of Central South University, Changsha, Hunan, China; ^3^ Department of Oncology, The Second Xiangya Hospital of Central South University, Changsha, Hunan, China

**Keywords:** soft tissue sarcoma, lncRNA, cuproptosis, signature, tumor immune microenvironment

## Abstract

**Background:** A crucial part of the malignant processes of soft tissue sarcoma (STS) is played by cuproptosis and lncRNAs. However, the connection between cuproptosis-related lncRNAs (CRLs) and STS is nevertheless unclear. As a result, our objective was to look into the immunological activity, clinical significance, and predictive accuracy of CRLs in STS.

**Methods:** The Cancer Genome Atlas (TCGA) and Genotype-Tissue Expression (GTEx) databases, respectively, provided information on the expression patterns of STS patients and the general population. Cuproptosis-related lncRNA signature (CRLncSig) construction involved the univariate, multivariate, and least absolute shrinkage and selection operator Cox regression analysis. The predictive performance of the CRLncSig was evaluated using a serial analysis. Further research was done on the connections between the CRLncSig and the tumor immune milieu, somatic mutation, immunotherapy response, and chemotherapeutic drug susceptibility. Notably, an *in vitro* investigation served to finally validate the expression of the hallmark CRLs.

**Results:** A novel efficient CRLncSig composed of seven CRLs was successfully constructed. Additionally, the low-CRLncSig group’s prognosis was better than that of the high-CRLncSig group’s based on the new CRLncSig. The innovative CRLncSig then demonstrated outstanding, consistent, and independent prognostic and predictive usefulness for patients with STS, according to the evaluation and validation data. The low-CRLncSig group’s patients also displayed improved immunoreactivity phenotype, increased immune infiltration abundance and checkpoint expression, and superior immunotherapy response, whereas those in the high-CRLncSig group with worse immune status, increased tumor stemness, and higher collagen levels in the extracellular matrix. Additionally, there is a noticeable disparity in the sensitivity of widely used anti-cancer drugs amongst various populations. What’s more, the nomogram constructed based on CRLncSig and clinical characteristics of patients also showed good predictive ability. Importantly, Real-Time Quantitative Polymerase Chain Reaction (RT-qPCR) demonstrated that the signature CRLs exhibited a significantly differential expression level in STS cell lines.

**Conclusion:** In summary, this study revealed the novel CRLncSig could be used as a promising predictor for prognosis prediction, immune activity, tumor immune microenvironment, immune response, and chemotherapeutic drug susceptibility in patients with STS. This may provide an important direction for the clinical decision-making and personalized therapy of STS.

## Introduction

The malignant tumor arising from mesenchymal tissues is known as soft tissue sarcoma (STS). It originates from mesenchymal tissue with distinctive differences in the site of occurrence, transformed cell types, clinical features, and histopathological characteristics of the various pathological types (Dickson et al., 2016). STS mainly occurs in children and adolescent groups, its incidence is low, and it is relatively rare, accounting for about only 1% of all malignancies in adults and about 7% of all malignancies in the pediatric population (Kapoor and Das, 2012; Gamboa et al., 2020). Even though STS is a relatively uncommon disease, its aggressive and metastatic nature usually leads to a poor prognosis for patients (Sachpekidis et al., 2019). Currently, radiation, chemotherapy, and surgical resection are used in conjunction to treat STS (von Mehren et al., 2020). Targeted and immunotherapy have also been suggested as treatments for STS in recent years (Yang et al., 2019). However, because of the sneaky symptomatology, delayed start of clinical symptoms, and quick progression of STS, the 5-year survival is only 63.9% (Chávez et al., 2019). Consequently, it is imperative to investigate brand-new, potential biomarkers to assist with the early detection of STS and its treatment.

The mineral element copper is essential throughout many biological processes, and it has long been considered a cofactor in the active sites for many metalloproteins only (Gou et al., 2021). However, emerging evidence suggests that patients with tumors had higher serum and tissue copper levels than people without tumors, indicating that copper may have a role in the onset and development of cancer (Babak and Ahn, 2021). Nevertheless, how excess copper plays a specific role in tumors remains inconclusive and requires further study. Cupropptosis, a fresh kind of programmed cell death that distinguishes from apoptosis, ferroptosis, pyroptosis, and necroptosis, has recently been proposed by Peter Tsvetkov et al. (2022). They discovered that copper might directly bind to lipid-acylated TCA cycle components, causing aggregation, dysregulation of associated proteins, and inhibiting the TCA cycle, which causes proteotoxic stress and ultimately results in cell death. And elesclomol is a promising copper ion carrier that exhibits tumoricidal effects through copper toxicity (O'Day et al., 2013). Together, these findings could offer a fresh perspective on the therapies for specific tumors.

A vast family of non-coding RNA molecules with a length larger than 200 nucleotides (nts) are considered to be long non-coding RNAs (lncRNAs). There are accumulating studies demonstrating that lncRNAs participate in the malignant processes of STS, such as the occurrence, development, and metastasis (Min et al., 2017). And their levels of expression can be utilized to predict patient therapy and prognosis (Hu et al., 2021). Meanwhile, significant numbers of studies have confirmed that programmed cell death-based lncRNA labeling can effectively individualize the prognosis of tumor patients. For instance, colon cancer prognosis prediction utility of ferroptosis-related lncRNA signature was demonstrated by Wu et al. (2021a). In addition, our previous study has proved that the cuproptosis-related lncRNA signature (CRLncSig) could use for the prognosis prediction of osteosarcoma (Liu et al., 2022). However, the relationship between cuproptosis-related lncRNAs (CRLs) and STS and their predictive value is not clear so far.

Thus, we aimed to use bioinformatics and *in vitro* experiments to systematically explore the prognostic performance, clinical relevance, and immune activity of CRLs in STS. And our findings will reveal the importance of CRLs in STS and provide a foundation for prognosis prediction, immunity characteristics identification, clinical chemotherapy, and immunotherapy in STS.

## Materials and methods

### Data collection and procession

The Cancer Genome Atlas (TCGA; https://www.cancer.gov/aboutnci/organization/ccg/research/structural-genomics/tcga) database was used to gather the expression patterns of the STS cohort as well as the accompanying clinical information, somatic mutation, and copy number variation data. After excluding individuals with incomplete survival information, we included a total of 260 STS patients. Since there was insufficient normal tissue sample in the TCGA database, we obtained 911 normal samples’ gene expression profiles from the Genotype-Tissue Expression (GTEx) database (https://www.gtexportal.org/home/). After the results extraction from the sources is completed, they will be preprocessed according to strict steps for reordering, normalization, and elimination of batch effects. Supplementary Table S1 displays the specific clinical features of the STS cohort.

### Extraction of cuproptosis-related genes in soft tissue sarcoma

We acquired a total of 10 cuproptosis-related genes (CRGs) for the prior investigation (Supplementary Table S2) (Tsvetkov et al., 2022). Using the R package “limma,” we first contrasted how these CRGs were expressed in STS versus normal tissues. Then, the somatic mutations of these 10 CRGs in STS were visualized using the package “Maftools.” Finally, the “Circos” package was used to map Ciros and clarify the distribution of chromosomal positions of the 10 CRGs.

### Screening of differential expressed cuproptosis-related genes in soft tissue sarcoma

The differences between the samples were visualized by using principal component analysis (PCA). To find differential expressed cuproptosis-related genes (DECRLs) between STS and normal samples, both differential expression analysis and Pearson co-expression analysis were implied. Differential expression analysis was performed by the “limma” package to identify the differential expressed lncRNAs (DElncRNAs) among STS and normal cohorts. The criterion for DElncRNAs was an adjusted *p*-value < 0.05 and |logFC| < 1.5. Heatmap and volcano plots were used to display DElncRNAs. Next, the R software was applied to calculate correlation coefficients based on the CRG and lncRNA expression files, and lncRNAs with |*R* ^2^| > 0.3 and *p*-value < 0.05 were defined as CRLs. Ultimately, the DECRLs were obtained for the intersection of DElncRNAs and CRLs.

### Identification of cuproptosis-related long non-coding RNAs related to the prognosis of soft tissue sarcoma

We chose the univariate cox analysis to evaluate the connection between the DECRLs and the STS’s prognosis to acquire the prospective prognostic CRLs for the subsequent signature construction. *p*-value < 0.05 was the requirement for predicting CRLs.

### Establishment of a cuproptosis-related long non-coding RNA signature

Both the training group (*n* = 130) and test group (*n* = 130) were created from the STS cohort (*n* = 260) by random selection. The signature CRLs of the candidates among the predetermined CRLs were found by the least absolute shrinkage and selection operator (LASSO) Cox regression analysis in the training group. The optimal CRLncSig was determined using multivariate Cox regression analysis with the candidates’ signature CRLs. Based on the observable coefficient and expression level of each CRL, the risk score for each STS patient was evaluated by: RiskScore = Σβ_i_*X_i_ (*β*_i_: coefficients of the gene i, X_i_: expression values of the gene i). With that, patients with STS were separated into low- and high-CRLncSig groups following the training cohort’s median risk score.

### Evaluation and validation of cuproptosis-related long non-coding RNA signature

To compare the overall survival (OS) of these two groups, a Kaplan-Meier (K-M) analysis was performed. While the association between risk score and the STS’s prognosis was visualized by the R package “pheatmap” to plot the risk score dispersion and patient survival situation. The receiver operating characteristic (ROC) curves for the 1-, 3-, and five- year OS were plotted using the R package “timeROC.” For further validation, the same analysis methods mentioned above were performed in the testing and whole cohort.

Furthermore, subgroup K-M analysis was employed to evaluate the CRLncSig’s reliability in light of various clinical features (including age, gender, cut-off margin status, metastatic status, and new oncology events). To test the independence of CRLncSig as a prognostic factor, we lastly conducted univariate and multifactor Cox regression analysis of the risk score and clinical traits.

### Genes with differential expression in two risk groups

Clustered heat map volcano plot was applied to display the DEGs between the low- and high-CRLncSig groups. On the DEGs with |logFC| > 0.585 and an adjusted *p*-value < 0.05, additional functional enrichment analysis was carried out.

### Gene ontology and the kyoto encyclopedia of genes and genomes analysis

Using the R package “clusterProfiler,” GO and KEGG functional enrichment analysis was carried out to access the biological functions of the differential gene (Wu et al., 2021b). GO analysis included GO biological process (BP), GO cellular compartment (CC) and GO molecular function (MF). The top 10 outcomes of the GO analysis and the top 20 outcomes of the KEGG enrichment analysis were represented in bubble plots.

### Protein-protein interaction network and friends analysis

To further identify the top ten hub genes in these DEGs between the two CRLncSig groups, PPI network analysis and Friends analysis were utilized. The DEGs between the low- and high-CRLncSig groups for the PPI network were uploaded to a string database with default parameters, and Cytoscape software was used to view the network (Doncheva et al., 2019). Then, the Friends analysis was conducted to screen the top ten hub genes using the R package “GOSemSim.” With K-M analysis and ROC curves, the prognostic value of each hub gene in STS was examined.

### Gene set enrichment analysis and gene set variation analysis

To investigate possible molecular paths between distinct STS CRLncSig groups, we performed GSEA and GSVA analyses. The GSEA analysis was applied using the “clusterProfiler” package relying on the KEGG gene set (c2.cp.kegg.v7.4.symbols.gmt) acquired from The Molecular Signatures Database (MSigDB, https://www.gsea-msigdb.org/gsea/msigdb). The top ten enrichment pathways in the low- and high-CRLncSig categories (*p*-value < 0.05) were chosen. The GSVA method, which is non-parametric and unsupervised, is frequently employed to access the differences in pathway activity. The GSVA was conducted by the “GSVA” package. Based on the GSVA data, the “limma” package was applied to assess the differences across the distinct risk categories. The clustered heat maps’ presentation was then limited to enrichment pathways with |logFC| > 0.15 and an adjusted *p*-value < 0.05.

### Estimation of the tumor microenvironment, immune checkpoints, immune cell infiltration, and stemness

Additionally, a serial bioinformatic approach was used to infer how the CRLncSig and immunological state are related. Initially, the tumor microenvironment (TME) component proportions, comprising stromal, immune, and ESTIMATE scores, were assessed using the ESTIMATE algorithm based on the R package “estimate”. Similar to this, the STS’s infiltration abundance of 28 tumor immune cells was evaluated by the single sample gene set enrichment analysis (ssGSEA) algorithm. Then, the associations between the immune cell infiltration and each signature CRLs and hub gene were explored using Pearson correlation analysis. Also, we contrasted the extracellular matrix (ECM) and possible immune checkpoint expressions between the groups with low and high CRLncSig. In addition, the link between the stemness of STS and CRLncSig score was evaluated using four stemness indices (EREG-mDNAsi, EREG-mRNAsi, mRNAsi, and mDNAsi).

### Association between distinct cuproptosis-related long non-coding RNA signature groups and clinical characteristics

A Chi-square test was performed to evaluate the variations in clinical features between the low- and high- CRLncSig groups and determine the link between clinical characteristics of STS and the new CRLncSig. The heat map displays the results. Age, gender, histological type, cut-off margin status, metastatic status, and new oncologic events are the clinical factors that were compared.

### Evaluation of immunotherapy response and drug susceptibility

We examined the variations in immunotherapy response and chemotherapy medication susceptibility between the various CRLncSig groups to aid the clinical treatment for STS. To figure out the association between the unique CRLncSig group with and without response to anti-CTAL4 and anti-PDL1 inhibitors, the Subclass Mapping (SubMap) algorithm was applied. Besides, the corresponding results were visualized utilizing the “pheatmap” and “ggpubr” R packages. To compare chemotherapeutic susceptibility, we calculated half of the maximum inhibitory concentration (IC50) using the R package “pRRophetic.” The Wilcoxon sign-rank test was employed to evaluate the difference in IC50 between the low- and high-CRLncSig groups. And twelve widely used chemotherapeutic medications, including Cisplatin, Cytarabine, Docetaxel, Doxorubicin, Pazopanib, Vinorelbine, Vorinostat, Erlotinib, Gefitinib, Lapatinib, and Metformin, were included in the investigation.

### Construction of nomogram and calibration curve

As a foundation for the clinical applicability of the fresh CRLncSig of overall survival (OS) prediction for patients with STS, column line plots on the basis of risk scores and clinical parameters were created. The nomogram may aid in estimating the 1-, 2-, and 3-year OS. The calibration curve was also used to show how closely the actual rates match the potential outcomes anticipated by the nomogram. The “rms” package was used to create the nomogram and calibration curves.

### Cell lines and cell culture

The Procell Life Science& Technology Co., Ltd., (Hubei, China). Provided the human liposarcoma cell line (SW872) for usage in research. The American Type Culture Collection’s (ATCC) human synovial sarcoma cell line (SW982) was purchased. Fenghui Biotechnology Co., Ltd., (Hunan, China) supplied the human skin fibroblast cell line (HSF). In addition to 10% fetal bovine serum (Gibco, United States) and 1% penicillin-streptomycin solution (NCM Biotech, China), all cell lines were grown in Dulbecco’s modified Eagle’s medium (Gibco, United States). The cell was cultured at 37°C in a 5% CO2-humidified atmosphere.

### Real-time quantitative polymerase chain reaction

Total cellular RNA was extracted using the RNA Express Total RNA Kit following the manufacturer’s instructions (M050, NCM Biotech, China). After that, cDNA synthesis was carried out using the Revert Aid First Strand cDNA Synthesis Kit (K1622, Thermo Scientific, United States). Hieff qPCR SYBR Green Master Mix (High Rox Plus) (11203ES, YEASEN Biotech Co., Ltd., China) was used to measure gene expression, and the 2^−ΔΔCT^ method was used to calculate the results. For normalization, GAPDH served as the internal standard. Supplementary Table S3 provides the used primer sequences.

### Statistical analysis

R (version 4.0.1) and GraphPad Prism (version 9.0.0) were used to carry out all statistical analyses for this investigation. For comparing the differences between the different groups, the student’s *t*-test or one-way analysis of variance were utilized. The Chi-square test was used to compare the clinicopathological parameters of the high-CRLncSig and low-CRLncSig groups. The Log-rank test was used in the KM survival analysis to compare the OS of two different groups. The Pearson correlation test served as the basis for the correlation analysis. A statistically significant difference was defined as a *p*-value of 0.05 or lower.

## Results

### Identification of differential expressed cuproptosis-related genes in soft tissue sarcoma

**FIGURE 1:**
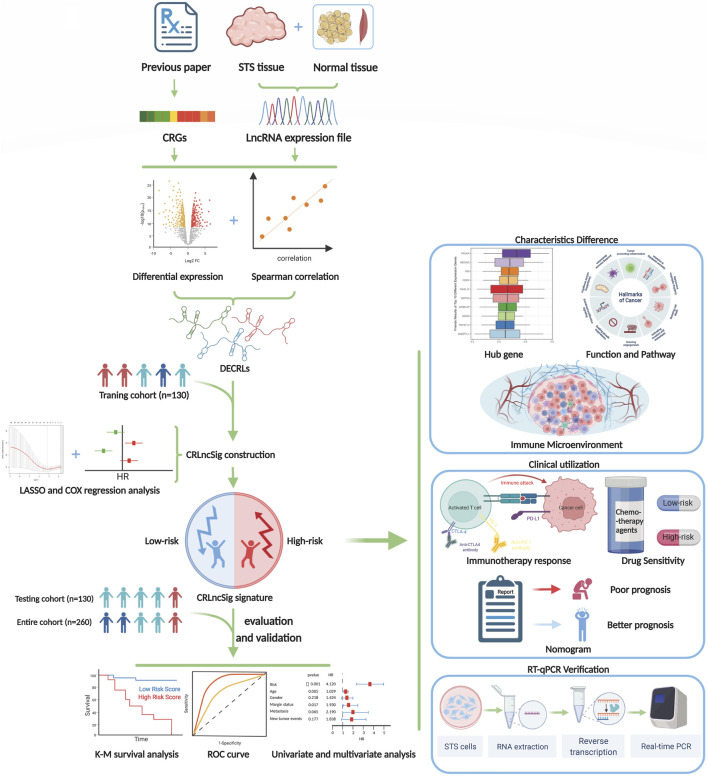
The flow diagram of our study.

**FIGURE 2:**
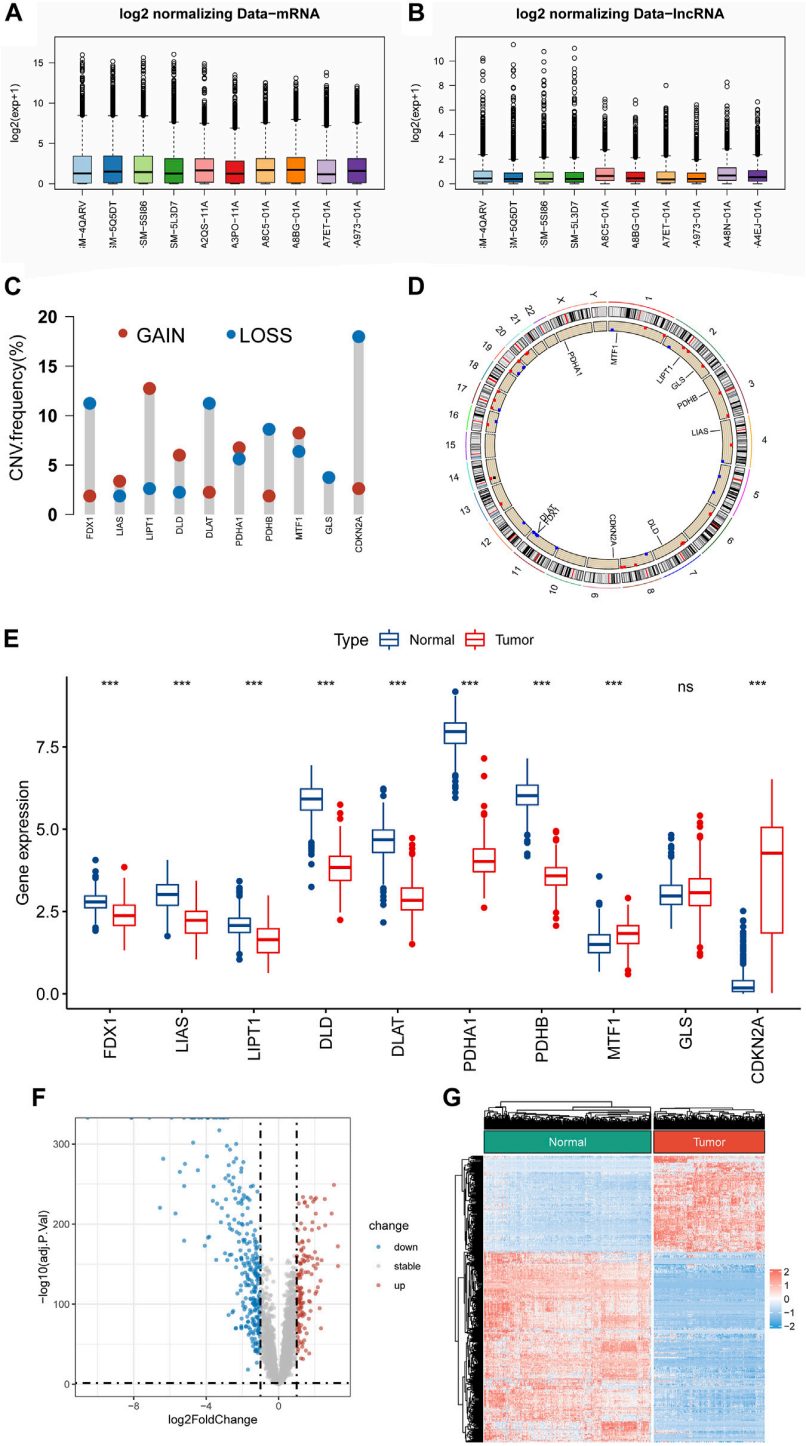
Identification of CRLs in STS. **(A,B)** Boxplots of mRNA and lncRNA expression profile after batch effect correction. **(C)** The frequencies of CNV gain and loss of CRGs. **(D)** The location of CRGs on chromosomes. **(E)** The expression level of CRGs between STS and normal tissue. **(F,G)** The volcano plot and heat map of the differentially expressed lncRNAs.

Figure 1 shows the flowchart of the present research. The mRNA and lncRNA expression profiles were normalized and batch corrected before analysis (Figures 2A,B). When we first measured the frequency of copy number variation (CNV) modifications, the result showed significant copy number amplification for LIPT1, DLD, and MTF1, while FDX1, DLAT, PDHB, and GLS exhibited significant copy number deletions (Figure 2C). And the location of CNV alteration on the chromosomes for each CRG is shown in Figure 2D. What’s more, except for GLS, the expression of almost all CRGs was significantly different between STS and normal tissue (Figure 2E). Unfortunately, no link was established between the altered CNV and the differential expression of CRGs. Subsequently, the PCA result based on the lncRNA expression demonstrated that the lncRNA expression profiles could clearly distinguish between the STS and normal samples (Supplementay Figure S1). As shown in Figures 2F,G, we identified 417 differentially expressed lncRNAs in STS according to the different analyses. And a total of 1103 lncRNAs were defined as CRLs through correlation analysis results (Supplementary Table S4). Finally, 145 differentially expressed CRLs were selected for the following signature construction (Supplementary Table S5).

### Constructing a cuproptosis-related long non-coding RNA prognostic signature

**FIGURE 3:**
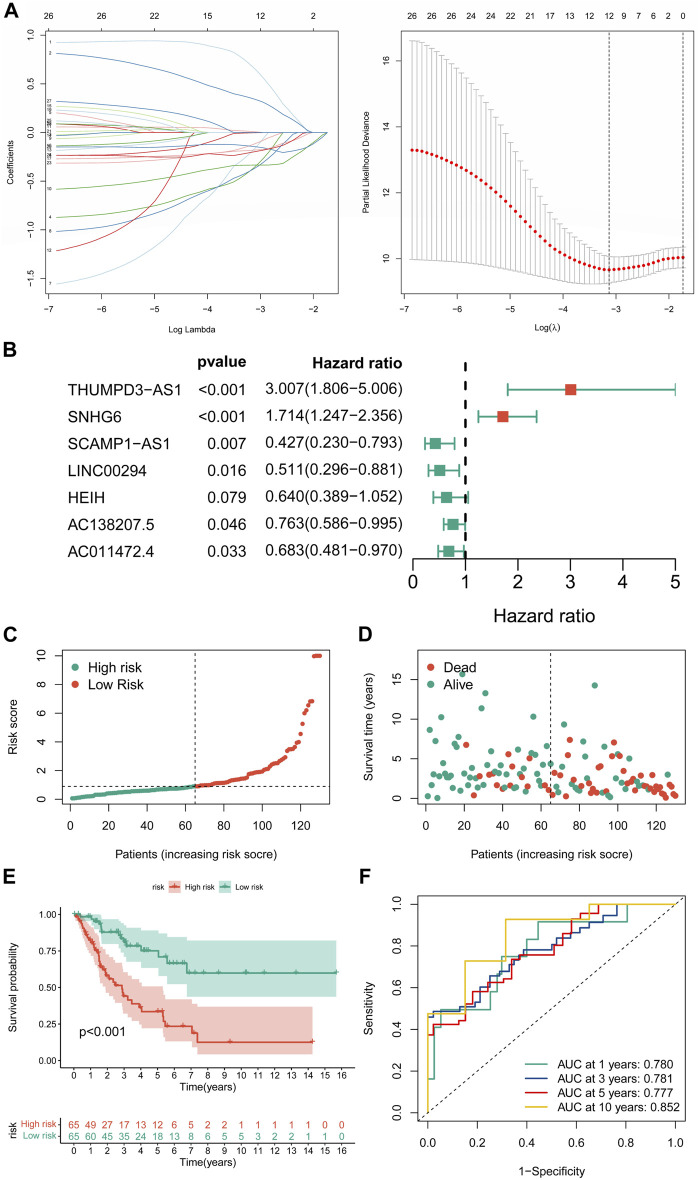
Construction of the novel CRLncSig. **(A)** The LASSO COX regression analysis. **(B)** The forest plot of the prognostic ability of the 7 CRLs included in CRLncSig. **(C,D)** The distribution of risk score and patient survival status. **(E)** The K-M survival curves of the novel CRLncSigin the training cohort. **(F)** Evaluate the prognostic performance of the risk score using time-dependent ROC in the training cohort.

Univariate Cox regression analysis revealed 27 CRLs linked to the prognosis of STS (Supplementary Table S6). After LASSO regression analysis, 12 candidate signature CRLs were screened out (Figure 3A). Subsequently, a novel CRLncSig was constructed based on multivariate Cox regression analysis, consisting of 7 CRLs, AC138207.5, THUMPD-AS1, LINC00294, SNHG6, AC011472.4, SCAMP1-AS1, and HEIH (Figure 3B; Supplementary Table S7). The CRLncSig was calculated as: risk score = THUMPD-AS1 ×1.100852728 + SNHG6 × 0.538933008—AC138207.5 × 0.27014193—LINC00294 × 0.671824437—AC011472.4 × 0.380716037—SCAMP1-AS1 × 0.850762533 + HEIH × 0.447005528. The distribution of risk scores and survival status implied that the deceased person was mostly concentrated in the high-CRLncSig group, suggesting that the STS’s prognosis may be responsive to the risk score (Figures 3C,D). Notably, the K-M analysis manifested that patients in the low-CRLncSig category seemed to have a more favorable survival than those in the high-CRLncSig group (*p* < 0.001, Figure 3E). Furthermore, good sensitivity and specificity in predicting the prognosis of STS were found through the new CRLncSig (Figure 3F).

### Evaluation and validation of the novel cuproptosis-related long non-coding RNA signature

**FIGURE 4:**
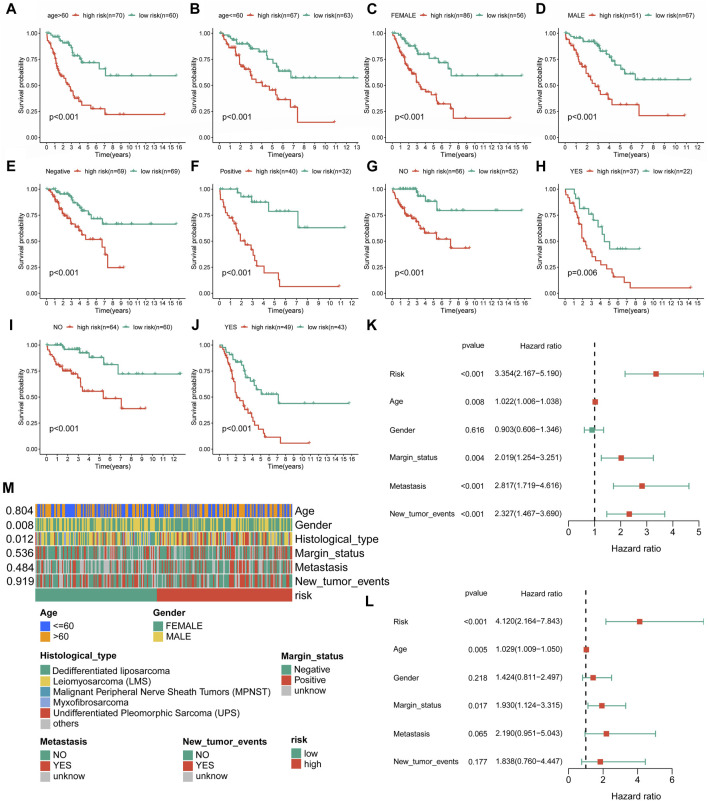
Independent prognostic value of the novel CRLncSig. **(A–J)** K–M survival curves is stratified by Age, Gender, Margin status, Metastasis status, and New tumor events between the distinct groups. **(K)** The univariate Cox regression analysis. **(L)** The multivariate Cox regression analysis. **(M)** Association between the risk scores and clinical features.

In further validation assessments, as expected, distribution plots, K-M analyses, and ROC curves for the training group and the whole cohort showed consistent results, which further demonstrated the feasibility of the novel CRLncSig (Supplementary Figures S2, S3). The innovative CRLncSig was found to be stable, as evidenced by subgroup survival analysis based on clinical features, which revealed that STS patients with lower risk scores had significantly increased OS regardless of clinical grouping (*p* < 0.001, Figures 4A–J). Additionally, the new CRLncSig was found to be an independent predictive risk factor for the STS group by univariate [hazard ratio (HR) = 3.354, 95% confidence interval (CI) = 2.167–5.190, *p* < 0.001] and multivariate (HR = 4.120, 95% CI = 2.164–7.843, *p* < 0.001) Cox analyses (Figures 4K–L). The interaction between the new CRLncSig and the clinicopathological features of STS was examined using a Chi-square test. And the heatmap revealed notable variations in the two CRLncSig groups’ gender and histology types (Figure 4M).

### Relationships between signature long non-coding RNA with cuproptosis in soft tissue sarcoma

**FIGURE 5:**
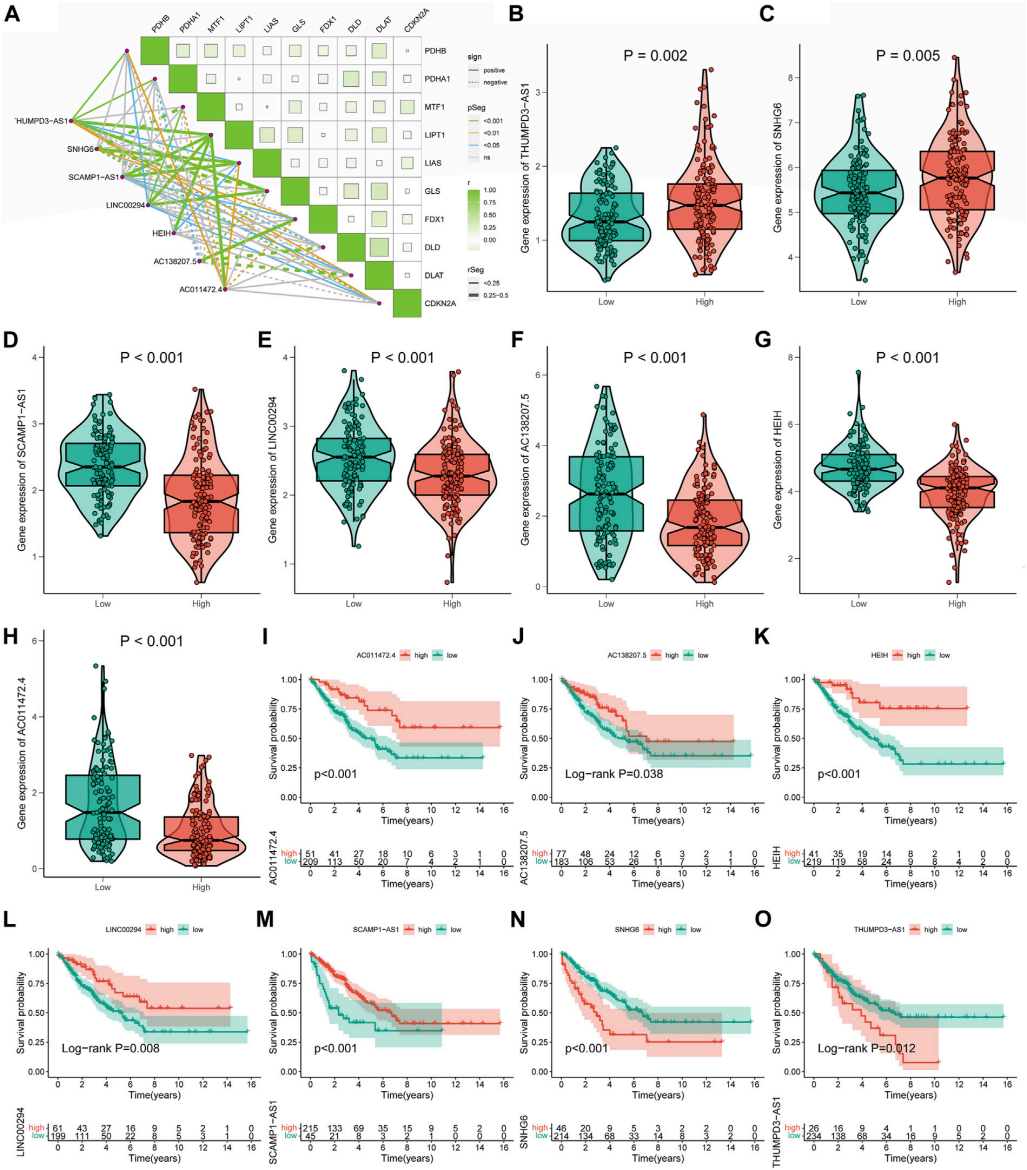
The relationships of seven signature CRLs with STS and cuproptosis. **(A)** Co-expression analysis of the seven signature CRLs with CRGs. **(B–H)** The expression level of seven signature CRLs in low-CRLncSig and high-CRLncSig groups. **(I–O)** The K-M survival curves for seven signature CRLs.

Co-expression analysis revealed an obvious co-expression correlation between the seven signature CRLs and CRGs (Figure 5A). Similarly, the expression of each signature CRLs showed a significant association (Supplementary Figure S4). When assessing the expression of labeled CRLs in the STS, in the high-CRLncSig group, it was observed that THUMPD-AS1 and SNHG6 represented higher expression, while AC138207.5, LINC00294, AC011472.4, SCAMP1-AS1, and HEIH were downregulated in the low-CRLncSig group (*p* < 0.001, Figures 5B–H). Likewise, the heatmap of testing, training, and entire groups displayed comparable outcomes (Supplementary Figure S5). Consist with the expression result, the K-M survival results indicated that the THUMPD-AS1 and SNHG6 exhibited a significant risk prognostic effect in STS, while AC138207.5, LINC00294, AC011472.4, SCAMP1-AS1, and HEIH showed a significant protective prognostic effect in STS (*p* < 0.05, Figures 5I–O). Hence, these results implied a potential prognostic value of these seven CRLs for STS.

### Genes with differential expression and functional enrichment analysis among different risk groups

**FIGURE 6:**
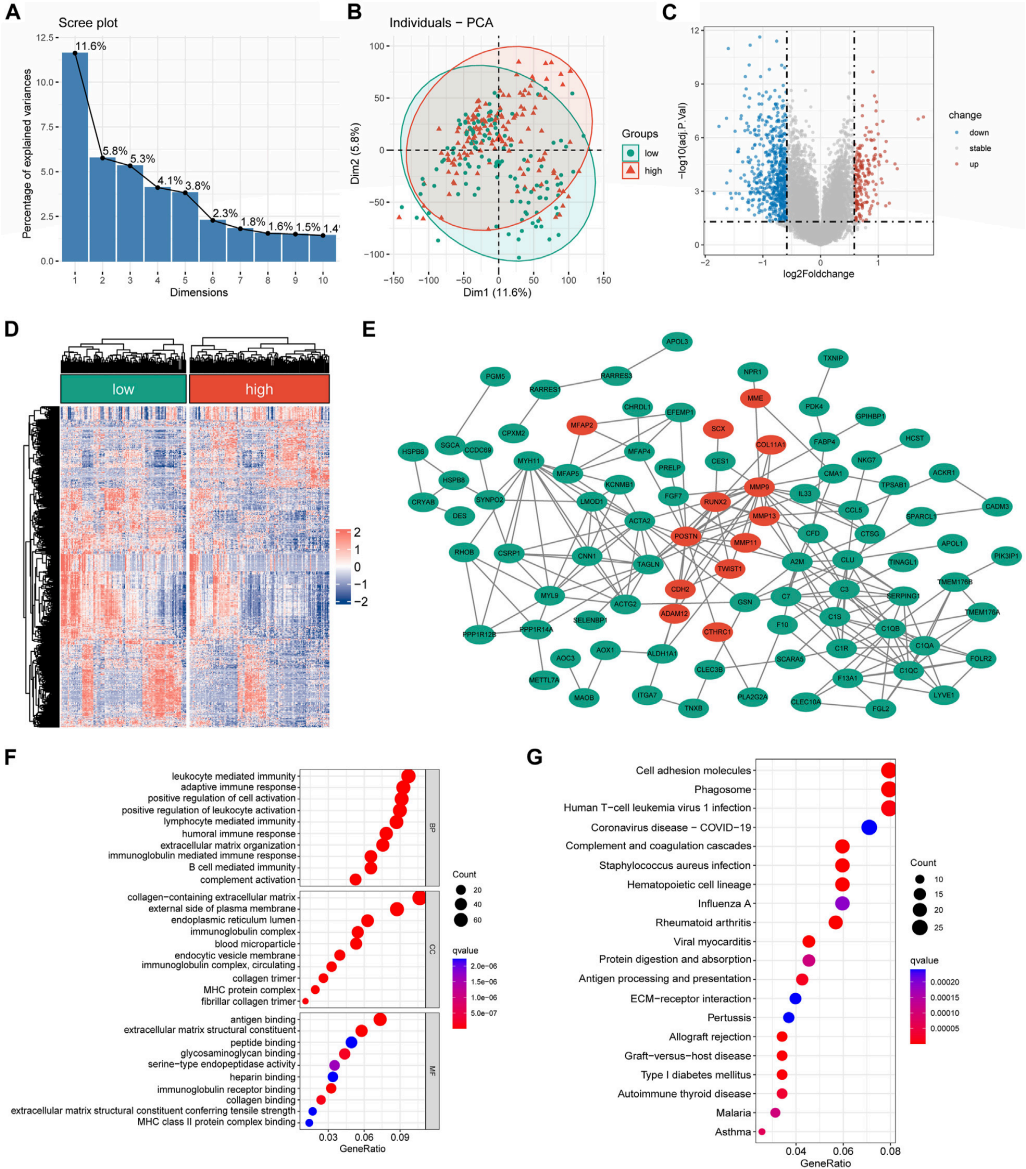
The differentially expressed and functional enrichment analysis between high- and low-CRLncSig groups. **(A)** The screen plot of PCA analysis is based on the differentially expressed gene between the low- and high-CRLncSig group. **(B)** PCA between high- and low-CRLncSig groups on all genes. **(C,D)** The volcano plot and heatmap of DEGs between the distinct risk group. **(E)** PPI network analysis of differential expressed gene. **(F,G)** The GO analysis and KEGG enrichment pathway analysis.

A substantial difference in the mRNA expression was found between the groups with low and high CRLncSig, according to differential analysis (Figures 6A,B). Under the screening criteria, 800 DEGs were discovered between various CRLncSig groups and their volcanoes (Figures 6C,D). Among them, 203 DEGs had increased regulation in the high-CRLncSig group, whereas 597 had decreased regulation. Figure 6E also displayed the PPI network. These DEGs were also found to be enhanced in immune response, B cell-mediated immunity, immunoglobulin-mediated immunity, immunoglobulin complex, MHC protein complex, MHC class II protein complex binding, etc., by the Go enrichment analysis (Figure 6F). Based on the KEGG database, we discovered that these DEGs were primarily concentrated in cell adhesion molecules, phagosomes, Human T-cell Leukemia Virus 1 infection, etc., (Figure 6G).

### Screening of cuproptosis-related hub genes in soft tissue sarcoma

**FIGURE 7:**
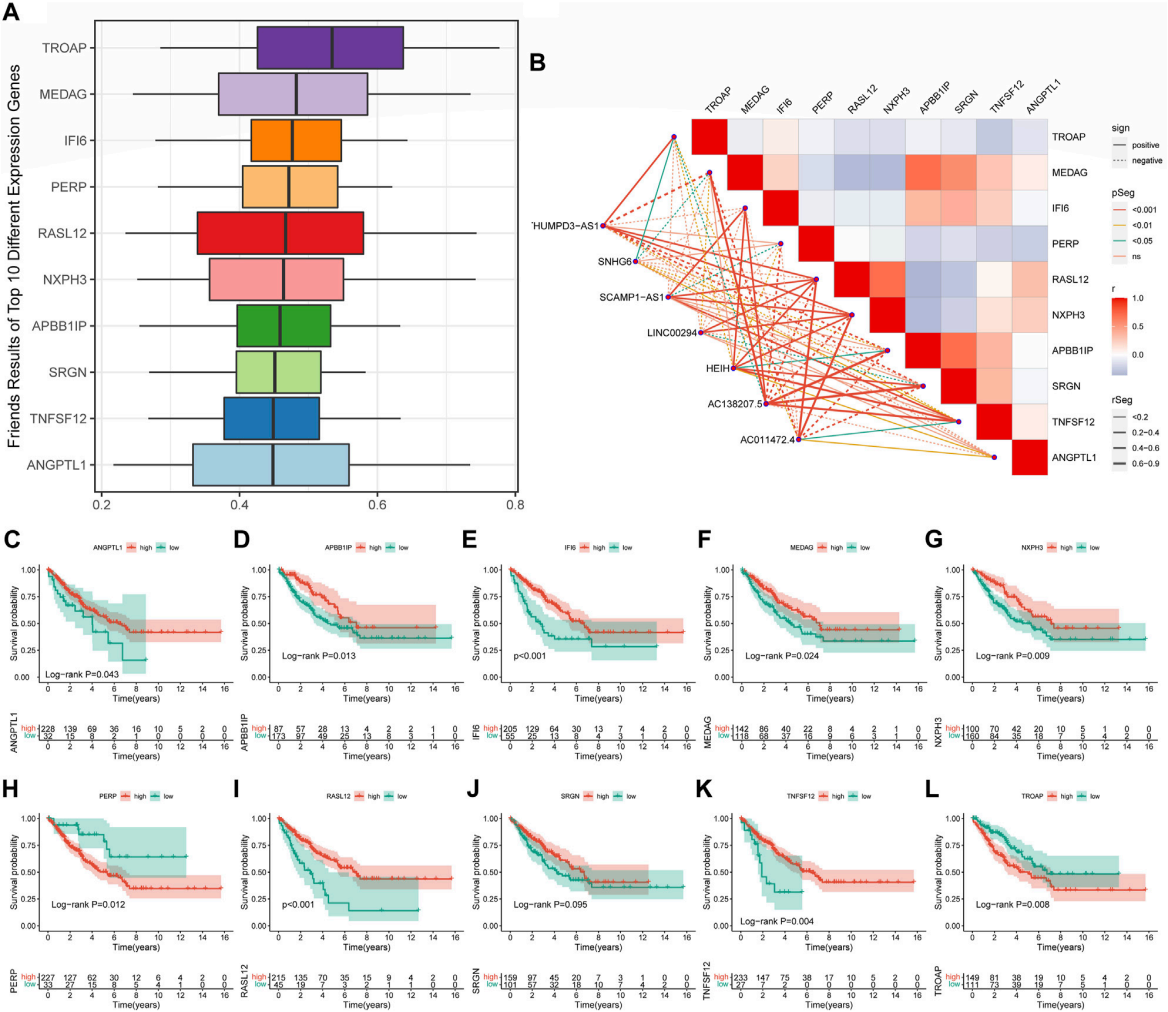
Screening cuproptosis-related hub genes. **(A)** The Friends analysis of GO-related genes. **(B)** The association between these ten hub genes and seven signature CRLs. **(C–L)** The K-M survival and ROC curve of these ten hub genes.

The Friends analysis revealed ten potential hub genes that may be involved in copper synapse formation (Figure 7A). And the co-expression results showed that these ten pivotal genes were closely associated with the signature CRLs (Figure 7B). As shown in Figures 7C–L, subsequent K-M survival analysis assessed the prognostic value of the identified key genes: ANGPTL1, APBB1IP, IFI6, MEDAG, NXPH3, RASL12, and TNFSF12 were positively associated with improved prognosis of STS, while elevated PERP and TROAP were associated with poor STS’s prognosis (*p* < 0.05). The above results suggested that these key genes may be potential prognostic biomarkers of STS and provide a reference for future STS biomarker studies.

### Exploration of the underlying pathways of cuproptosis-related long non-coding RNA signature

**FIGURE 8:**
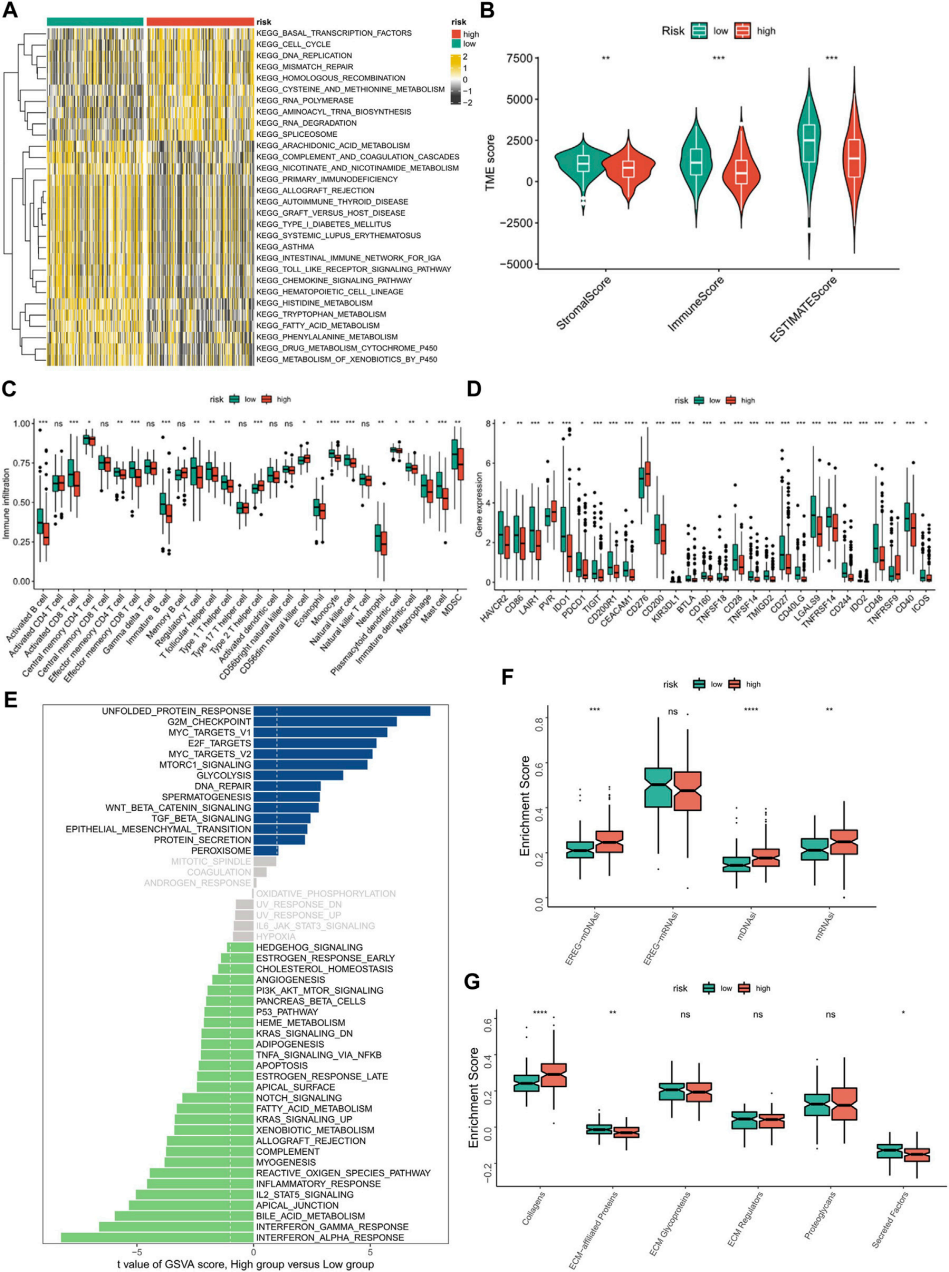
Evaluation of tumor microenvironment, immune cell infiltration, and immune checkpoint genes in distinct groups. **(A)** The heatmaps of GSVA showed signaling pathways between different risk groups. **(B)** Comparison of TME score between the high- and low-CRLncSig groups. **(C)** Differences in the infiltration of immune cells between the distinct risk groups. **(D)** The expression level of immune checkpoint genes between the high- and low-CRLncSig groups. **(E)** Differences in signaling pathways between different risk groups. **(F)** The differences in stemness index between the high- and low-CRLncSig groups **(G)** The different extracellular matrix components between the high- and low-CRLncSig groups.

According to Supplementary Figure S6, the pathways of CRLncSig in the high-CRLncSig group were primarily enriched in several tumor-related pathways, namely the cell cycle, DNA replication, ECM receptor interaction, pathway in cancer, TGF-beta signaling route, and Wnt signaling pathway. In contrast, CRLncSig pathways were primarily focused on the complement and coagulation cascades, cytokine-cytokine receptor interaction, and chemokine signaling pathway in the low-CRLncSig group. Encouragingly, the heatmap of GSVA showed similar results (Figures 8A,E). Based on the GSVA outcomes, the pathways of CRLncSig in the high-CRLncSig cohort were also enriched in the cell cycle and DNA replication, whereas the pathways of CRLncSig in the low-CRLncSig STS cohort were primarily enriched in immune-related pathways, along with chemokine signaling pathway, primary immunodeficiency, and Toll-like receptor signaling pathway. Together, these findings showed that the new CRLncSig was related to STS carcinogenesis and tumor immunity.

### Associations of cuproptosis-related long non-coding RNA signature with tumor microenvironment and immune cell infiltration

TME and immune cell infiltration were examined due to the variations in molecular biological processes and immune-related pathways between the two CRLncSig groups. Primarily, the ESTIMATE outcomes manifested that the low-CRLncSig group appeared to have greater stromal, immune, and estimate scores than those in the high-CRLncSig group (Figure 8B). Additionally, the low-CRLncSig group’s immune cells almost universally displayed better infiltration abundance as compared to the proportion of immune cells that were infiltrated in STS (Figure 8C). Also, the low-CRLncSig group showed additional immune checkpoint expression (Figure 8D). The correlation analysis also revealed that these signature CRLs and hub genes were positively correlated with immune cell infiltration (Supplementary Figure S7). The indices of mDNAsi, mRNAsi, and EREG-mDNAsi were considerably greater in the high-CRLncSig group, indicating that the high-CRLncSig group was related to higher stemness in STS, based on an analysis of the association between the stemness of STS and CRLncSig score (Figure 8F). And it also implied the high-CRLncSig group was more aggressive. The extracellular matrix of the two groups was further analyzed, and it was discovered that the high-CRLncSig group had much more collagen than the low-CRLncSig group did, while the low-CRLncSig group had more ECM-related proteins and secreted factors (Figure 8G). Altogether, these findings showed that the STS cohort with a higher CRLncSig score had a poor immune status, higher tumor stemness, and higher collagens in ECM, which may account for the prognosis difference of patients in the distinct groups.

### Tumor mutation status between two distinct cuproptosis-related long non-coding RNA signature groups

**FIGURE 9:**
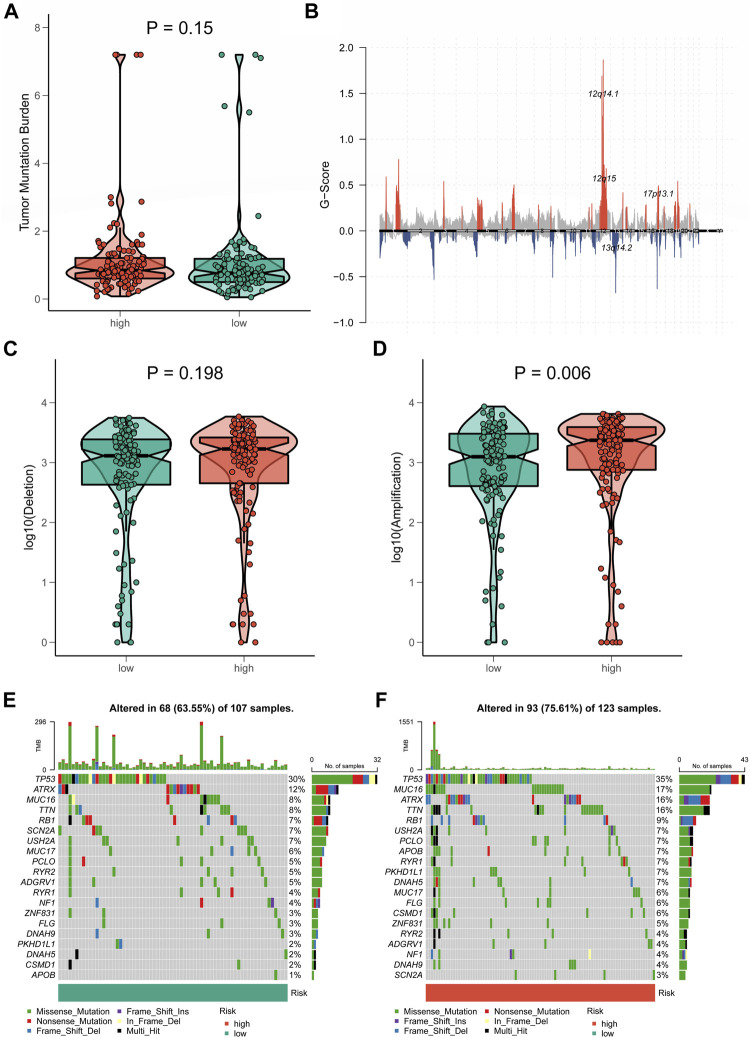
Tumor mutation status among different risk groups. **(A)** TMB in the high- and low-CRLncSig groups. **(B)** The genome-wide gene CNV of STS. **(C,D)** The difference in CNV loss and gain between the distinct risk groups. **(E,F)** The differences in mutations between distinct risk groups (the top 20 mutated genes).

A growing body of research revealed that TMB was closely associated with tumor immunotherapy (Wang et al., 2019). However, significant differences in TMB scores and patterns of chromosomal alterations between CRLncSig groups had not been observed (*p* = 0.15, Figures 9A,B). No discernible difference was found in the frequency of copy-number loss between the two CRLncSig groups (*p* = 0.198, Figure 9C), whereas copy-number expansion was more common in the high-CRLncSig group than in the low-CRLncSig group (*p* < 0.05, Figure 9D). Additionally, Figures 9E,F displayed the differences in somatic mutation distribution across the two separate CRLncSig groups, and these waterfall plots displayed the detailed mutation spectrum of the top 10 mutated genes in the high-CRLncSig and low-CRLncSig groups.

### Cuproptosis-related long non-coding RNA signature predicts efficacy of immunotherapy response and chemotherapy susceptibility

**FIGURE 10:**
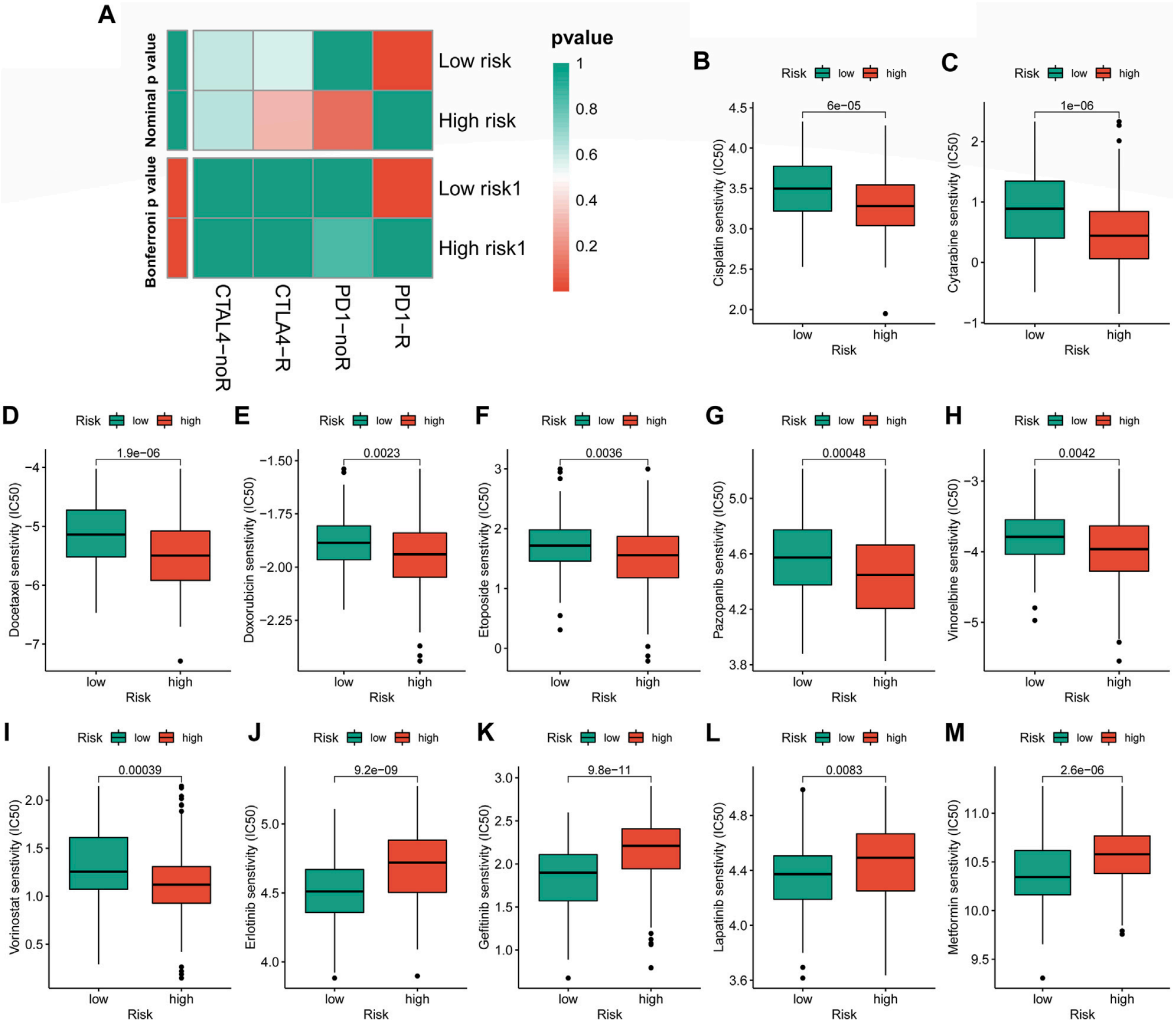
Evaluation of immunotherapy response and chemotherapy sensitivity in STS with a different risk score. **(A)** The therapeutic responses to immune checkpoint inhibitors in STS patients. **(B–M)** The differences in estimated IC50 values of 12 representative drugs between the high- and low-CRLncSig groups.

In the case of immunotherapy, the low-CRLncSig group achieved higher responses to PD-1 and CTLA-4 inhibitors (Figure 10A). The IC50 of 12 chemotherapy drugs was different between these two groups (*p* < 0.05), according to Figures 10B–M, which also showed the relationship between risk scores and sensitivity to commonly used anti-cancer medications. Patients in the high-CRLncSig group had lower IC50 values for the drugs cisplatin, cytarabine, docetaxel, doxorubicin, pazopanib, vinorelbine, and vorinostat (Figures 10B–I), but had higher IC50 values for erlotinib, gefitinib, lapatinib, and metformin than those in the low-CRLncSig group (Figures 10J–M).

### Establishment of the nomogram

**FIGURE 11:**
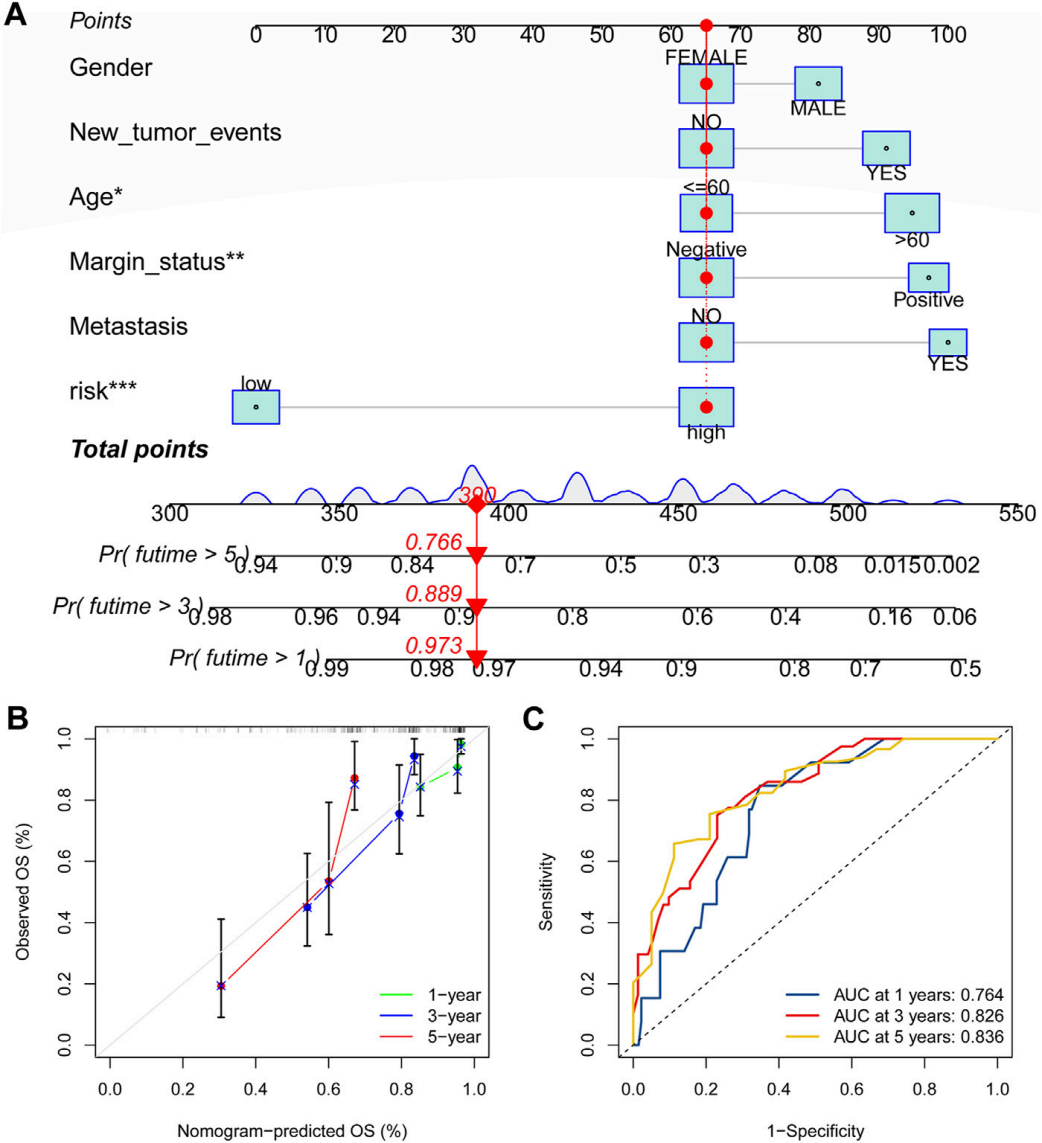
Construction of the nomogram for predicting overall survival for STS. **(A)** Nomogram based on the novel CRLncSigin for predicting 1-, 3-, and 5-year overall survival of STS. **(B)** Calibration curves of the nomogram. **(C)** The area under the time-dependent ROC curves for nomogram at 1-, 3-, and 5-year.

As shown in Figure 11A, the nomogram created using the CRLncSig risk score and other clinical traits made it possible to accurately forecast OS of 1-, 3-, and 5- years. And the ROC curve exhibited that this nomogram offered great prognostic prediction accuracy for patients with STS. The nomogram’s area under the curve (AUC) values for years 1, 3, and 5 were 0.788, 0.758, and 0.739 (Figure 11B). Furthermore, the calibration curve showed that the nomogram’s predictions for 1-, 3-, and 5-year OS under the ideal model worked well (Figure 11C). The above outcomes manifested that the nomogram had strong predictive power.

### Verification of the signature long non-coding RNA expression

**FIGURE 12:**
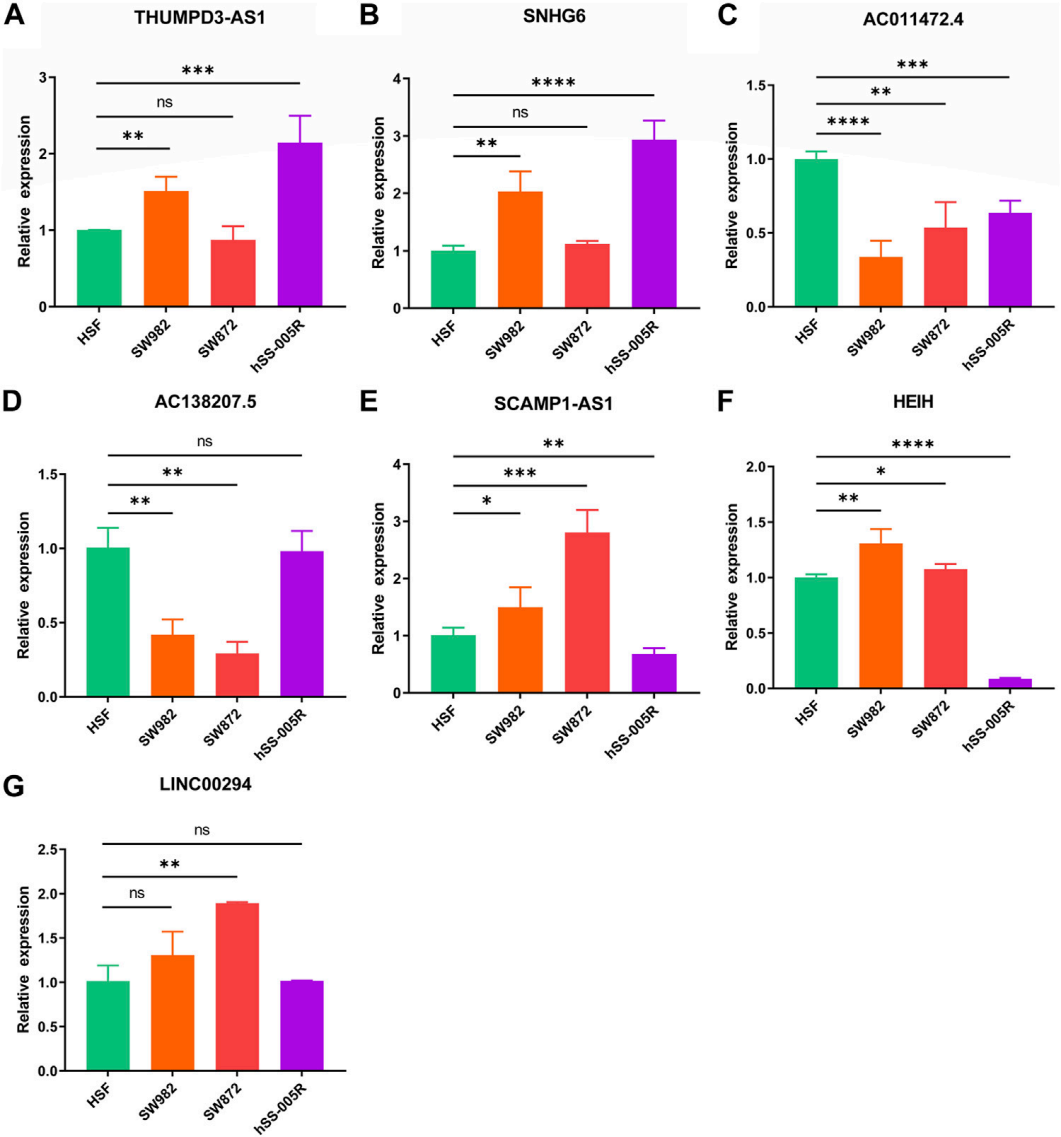
Verification of the expression of signature CRLs in OS cell lines. **(A)** THUMPD3-AS1. **(B)** SNHG6. **(C)** AC011472.4. **(D)** AC138207.5. **(E)** SCAMPA-AS1. **(F)** HEIH. **(G)** LINC00294. *p < 0.05, **p < 0.01, ***p < 0.001, ****p < 0.0001.

Ultimately, it was discovered that THUMPD-AS1 and SNHG6 were enhanced in the STS cell lines (SW982, SW872, and hSS-005R), particularly in SW982 and hSS-005R, when RT-qPCR was utilized to confirm these signature CRLs’ expression in the STS cell lines (Figures 12A,B). Contrary, compared with the normal cell lines, AC011472.4 and AC138207.5 were reduced in the STS cell lines (Figures 12C,D). In addition, SCAMPA1-AS1 and HEIH were overexpressed in SW982 and SW872 cell lines while dramatically decreasing in hSS-005R (Figures 12E,F). And LINC00294 exhibited an increasing trend in the STS cell lines, especially in the SW872 cell line (Figure 12G).

## Discussion

STS is a rare group of soft tissue malignancies that include a variety of histological subtypes and can occur in any anatomic location (Agulnik et al., 2017). Due to the rarity of STS and its high histological subtype heterogeneity, a bottleneck has been encountered in improving the prognosis of STS. Furthermore, early detection, diagnosis, and therapy are essential components in enhancing the clinical survival prognosis for STS. Currently, cuproptosis offers a potential insight for forecasting the prognosis of tumor patients into the prognosis prediction of the tumor, according to mounting data. A solid basis for tumor treatment has already been laid by a previous study that identified a cuproptosis-related gene signature associated with clear cell renal cell carcinoma and suggested that it might function as a possible prognostic predictor for the disease (Bian et al., 2022). Additionally, cuproptosis associated lncRNA had good prediction ability for osteosarcoma. However, studies examining the connection between CRLs and STS were still infrequent.

In the present study, a novel CRLncSig consisting of seven CRLs was successfully constructed, which could be used as a promising tool for assessing prognosis prediction, immune status, immunotherapy response, and chemotherapy in STS. Initially, 1103 CRLs in STS were screened by analysis of cuprotosis-related genes and expression files of STS. Next, the univariate Cox analysis reflected that 27 CRLs were related to the prognosis of STS. Following these prognostic CRLs, seven CRLs were screened for signature construction using the LASSO and multivariate Cox regression analysis. Moreover, the K-M analysis and ROC curve manifested that the novel CRLncSig had a robust prognostic, predictive ability. Additionally, the internal validation, clinical subgroups survival analysis, and independent analysis further proved that the novel CRLncSig had excellent, stable, and independent prognostic and predictive utility for STS.

Furthermore, the Friend analysis revealed that the pivotal genes ANGPTL1, APBB1IP, IFI6, MEDAG, NXPH3, RASL12, TNFSF12, PERP, and TROAP might serve as promising biomarkers. Remarkably, some earlier studies have shown the significance of these hub genes in cancers. For instance, ANGPTL1 has been proven to inhibit tumor metastasis in several cancers. According to Ge et al. (2021), the APBB1IP could be used as a predictive biomarker in malignancies, and its expression is relevantly linked with the tumor immune microenvironment. IFI6, also known as G1P3, is a mitochondrial localized antiapoptotic protein that has been shown to promote the metastatic ability of breast cancer cells through mtROS (Cheriyath et al., 2018). Consistently, Dai et al. (2021) also found MEDAG to be a protective factor for the prognosis of STS, further confirming the accuracy of the CRLncSig. And RASL12 exhibited a diminished expression in lung adenocarcinoma, and its downregulation was positively correlated with better OS (Li et al., 2021). Besides, the overexpression of TNFSF12 has been proven to promote apoptosis in gefitinib-resistance cell lines (Li et al., 2018). In contrast, TROAP was anticipated to be a new target for glioma therapy since it could accelerate the malignant growth of gliomas by activating the Wnt/β-Catenin signaling pathway (Zhao et al., 2021). According to our findings and other research, these hub genes may be essential in STS and serve as a foundation for upcoming biomarker studies.

We used GSEA and GSVA to further research the molecular mechanism of how the new CRLncSig impacts the prognosis of STS. The findings showed that pathways implicated in carcinogenesis and progression, such as the cell cycle, TGF-beta signaling system, and Wnt signaling route, were notably enriched in the CRLncSig pathways in the high-CRLncSig category. As critical signaling pathways that are associated with tumorigenesis, previous studies have established that these pathways have a vital role in promoting malignant phenotype in STS. The abnormal cell cycle regulation is a vital cancer hallmark (Hanahan and Weinberg, 2011), and it has been proven that the disruption of cell cycle monitoring and proliferation mechanisms is the leading cause of the proliferation and specific phenomena of tumor cells (Lv et al., 2019). In addition to being linked with the possession of embryo growth and tissue homeostasis, the Wnt signaling pathway is also linked to the development of cancer (Zhan et al., 2017). And TGF-beta is a multifunctional regulator of cell growth and differentiation (Wang et al., 2003). Chen et al. (2019) discovered that GDF15 might control the TGF-beta signaling pathway to help osteosarcoma’s ability to invade and migrate. Additionally, earlier research has shown that immunological status has a significant impact on the prognosis of STS (Lin et al., 2021). The immunological state may be linked to a better prognosis in the low-CRLncSig group, given this study’s finding that the pathways of CRLncSig in the low-CRLncSig STS cohort were largely enriched in immune-related pathways. The aforementioned findings and studies thus show that these tumor-related pathways and immunological states may be related to new CRLncSig in STS, but the specific relationship needs to be further investigated.

Different immunological states may influence the clinical course of the same type of tumor in different ways (Zhou et al., 2021). In comparison to the high-CRLncSig group patients, the low-CRLncSig group patients had better stromal, immunological, and TME cells. And this is in line with the outcomes of several earlier STS research (Huang et al., 2021; Qi et al., 2022). Similarly, the abundance of lots of immune cells infiltrating the body involving natural killer cells (NK cells), activated CD8 T cells, mast cells, etc., was higher in the low-CRLncSig group than in the high-CRLncSig group. As part of innate immunity, NK cells exhibit a suppression effect in the development of tumors (Yamamoto et al., 2018). Meanwhile, the activated CD8 T cells can directly kill tumor cells or mediate cytotoxic antitumor immune responses by producing granzyme or perforin, IFN-γ, and tumor necrosis factor (Hadrup et al., 2013; Wang et al., 2021). Additionally, tumor development and metastasis are tightly correlated with tumor stemness. High stemness STS were reported to have a worse prognosis and be more likely to metastasize. Low immunological infiltration of immune killer cells and more immunosuppressive cells are signs of poor prognosis, whereas patients with low stemness showed stronger immune status and better prognoses. Cancer frequently exhibits dysregulation and disruption of the ECM, and the ECM is intimately linked to the development of cancer. Moreover, collagen matrix has been shown to significantly enhance the metastatic potential of tumors, and collagen degradation could enhance the immune microenvironment. According to the outcomes of this study, which are consistent with those of earlier studies, the high-CRLncSig group’s stem cells had higher stemness, more collagen matrix, and worse immune status. Combined with the above analyses and previous reports, The various prognoses of STS patients with different risk scores may be explained by considerable disparities in the immunological state, stemness, and ECM of STS patients in diverse CRLncSig risk categories. And there is also a correlation between the ECM, stemness, and immune status.

In addition, we noticed that the CRLncSig risk scores and the expression of most immune checkpoint genes were inversely associated. And it has been proposed that immune checkpoint expression may be a reflection of the clinical outcome of immunotherapy that targets immunological checkpoints (Zheng et al., 2021). Immunotherapy that targets immunological checkpoints (PD-1 and CTLA-4) has recently become a potential treatment option for a variety of malignancies (Zhou et al., 2017; Chen et al., 2021). However, each tumor exhibits a different response to immunotherapy (Zou et al., 2016). It is interesting to note that this study’s findings suggested that patients of the low-CRLncSig category reacted better to PD-1-targeted immunotherapy, bringing renewed hope for personalized immunotherapy. Meanwhile, due to drug resistance and heterogeneity, STS is relatively insensitive to various chemotherapeutic agents, resulting in limited benefits in chemotherapy (Chang et al., 2018). To examine the response to chemotherapy in various CRLncSig groups, we chose 12 regularly used anti-cancer medicines. Higher sensitivity to cisplatin, cytarabine, docetaxel, doxorubicin, pazopanib, vincristine, and vorinostat was shown in high-CRLncSig group patients. The low-CRLncSig group patients, however, responded favorably to the medications erlotinib, gefitinib, lapatinib, and metformin. Altogether, this study provided an approach to optimize the combination regimen of immunotherapy and chemotherapy based on the novel CRLncSig, providing practical information for the individualized treatment of STS.

Furthermore, we finally detected the expression of CRLs in the signature using RT-qPCR and discovered that the expression of these CRLs varied significantly amongst the STS cell lines. And it has been shown that several CRLs had important roles in a variety of cancers. Among them, THUMPD3-AS1 may have an impact on Non-Small Cell Lung Cancer cell growth and self-renewal through controlling miR-543 and ONECUT2 (Hu et al., 2019). And the expression of LINC00294 is downregulated in glioma, thereby targeting miR-1278 to promote NEFM and inhibit glioma cell proliferation (Zhou et al., 2020). Meanwhile, SNHG6 expression is increased in osteosarcoma and is a new risk prognostic biomarker for osteosarcoma (Ruan et al., 2018). In contrast, AC011472.4 was discovered as a new lncRNA biomarker linked to the poor OS in colorectal cancer patients (Chu et al., 2020). A significant functional role for HEIH in the occurrence and growth of malignancies has been demonstrated in earlier research. According to Wan et al. (2020), HEIH could improve the ability of cholangiocarcinoma cells to proliferate, migrate, and invade by controlling miR-98-5p and HECTD. However, the roles of SCAMP1-AS1 and AC138207.5 in tumorigenesis and progression are still poorly understood. Thus, these findings suggested that these signature CRLs may play a significant predictive role in the STS cohorts’ prognosis in the various CRLncSig groups and may serve as a useful benchmark for related studies.

Despite the robust findings, there are still several issues with this study’s limitations that need to be resolved. First, because other databases lack information on the same signed CRL, external validation cannot be performed. Although the RT-qPCR may be regarded as a validation, further verification by more clinical cohorts is still needed in the future. In addition, extensive and relevant *in vivo* and *in vitro* experimental validation is necessary to understand the precise function of the signature CRLs in the development of STS and to support our findings.

## Conclusion

In summary, this study comprehensively analyzed the CRLs in STS and confirmed the predictive efficacy of the novel CRLncSig on the prognosis of STS. Moreover, the novel CRLncSig was closely relevant to the immune activity, tumor immune microenvironment, immune response, and chemotherapeutic drug susceptibility. This could lead to the development of novel techniques for the clinical prognostic prediction and personalized therapy of STS patients.

## Data Availability

The original contributions presented in the study are included in the article/Supplementary Material, further inquiries can be directed to the corresponding authors.

## Glossary

STS: soft tissue sarcoma
CRLs: cuproptosis-related lncRNAs
TCGA: the cancer genome atlas
GTEx: genotype-tissue expression
CRLncSig: cuproptosis-related lncRNA signature
RT-qPCR: real-time quantitative polymerase chain reaction
CRGs: cuproptosis-related genes
PCA: principal component analysis
DElncRNAs: differentially expressed lncRNAs
DECRLs: differential expressed cuproptosis-related genes
GO: gene ontology
KEGG: kyoto encyclopedia of genes and genomes
BP: biological process
CC: cellular compartment
MF: molecular function
PPI: protein-protein interaction
K-M: Kaplan-Meier
GSEA: gene set enrichment analysis
GSVA: gene set variation analysis
MSigDB: molecular signatures database
TME: tumor microenvironment
ssGSEA: single sample gene set enrichment analysis
ECM: extracellular matrix
SubMap: subclass mapping
IC50: maximum inhibitory concentration
OS: overall survival
ATCC: american type culture collection
HSF: human skin fibroblast
CNV: copy number variation
AUC: area under the curve
NK cells: natural killer cells
