# Supplementary figures and images for: Comprehensive analysis of a novel cuproptosis-related lncRNA signature associated with prognosis and tumor matrix features to predict immunotherapy in soft tissue carcinoma
Source: Front Genet. 2022 Dec 7;13:1063057. doi: 10.3389/fgene.2022.1063057 (PMC9768346; doi:10.3389/fgene.2022.1063057)

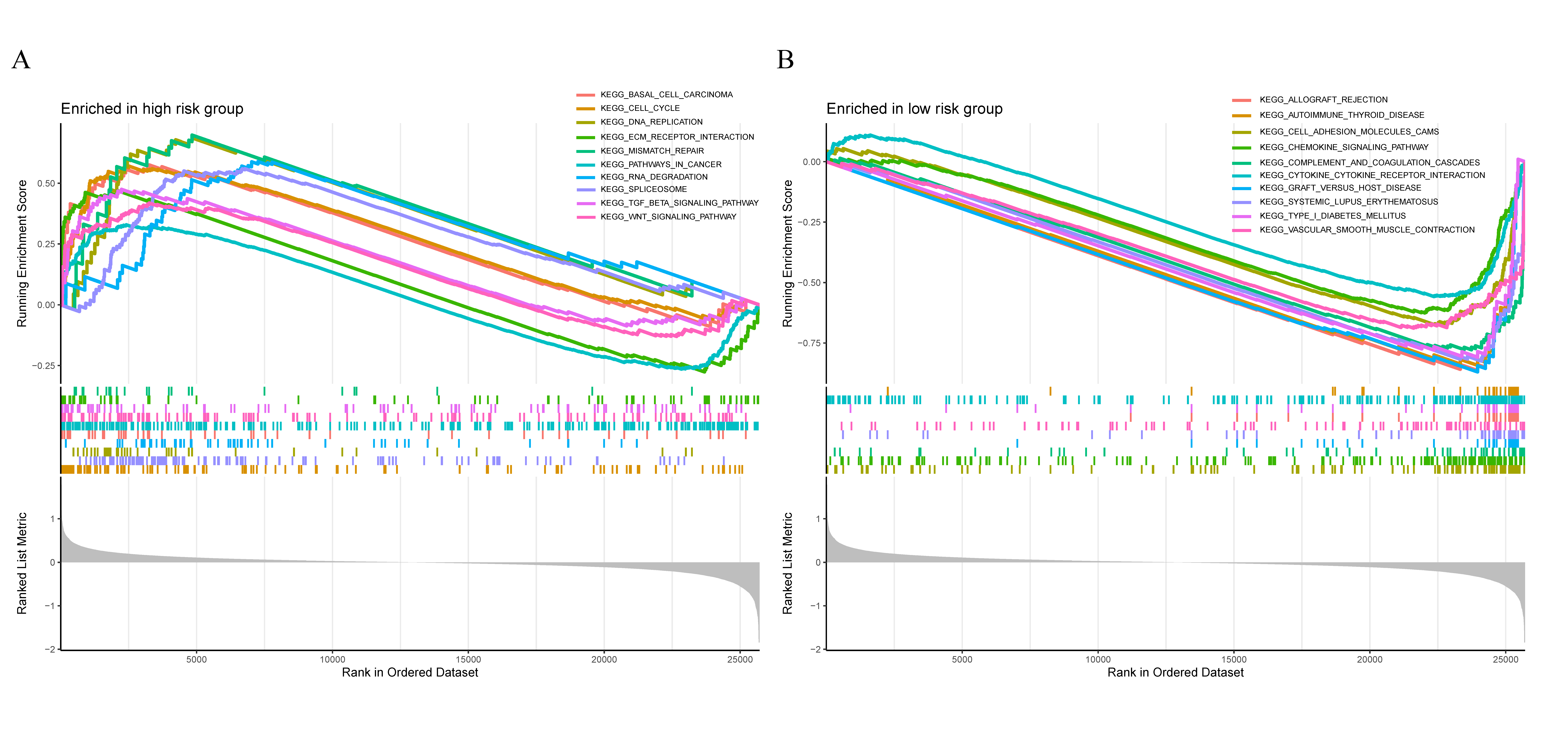

Supplement: Supplementary file 1 [file Image6.TIF]

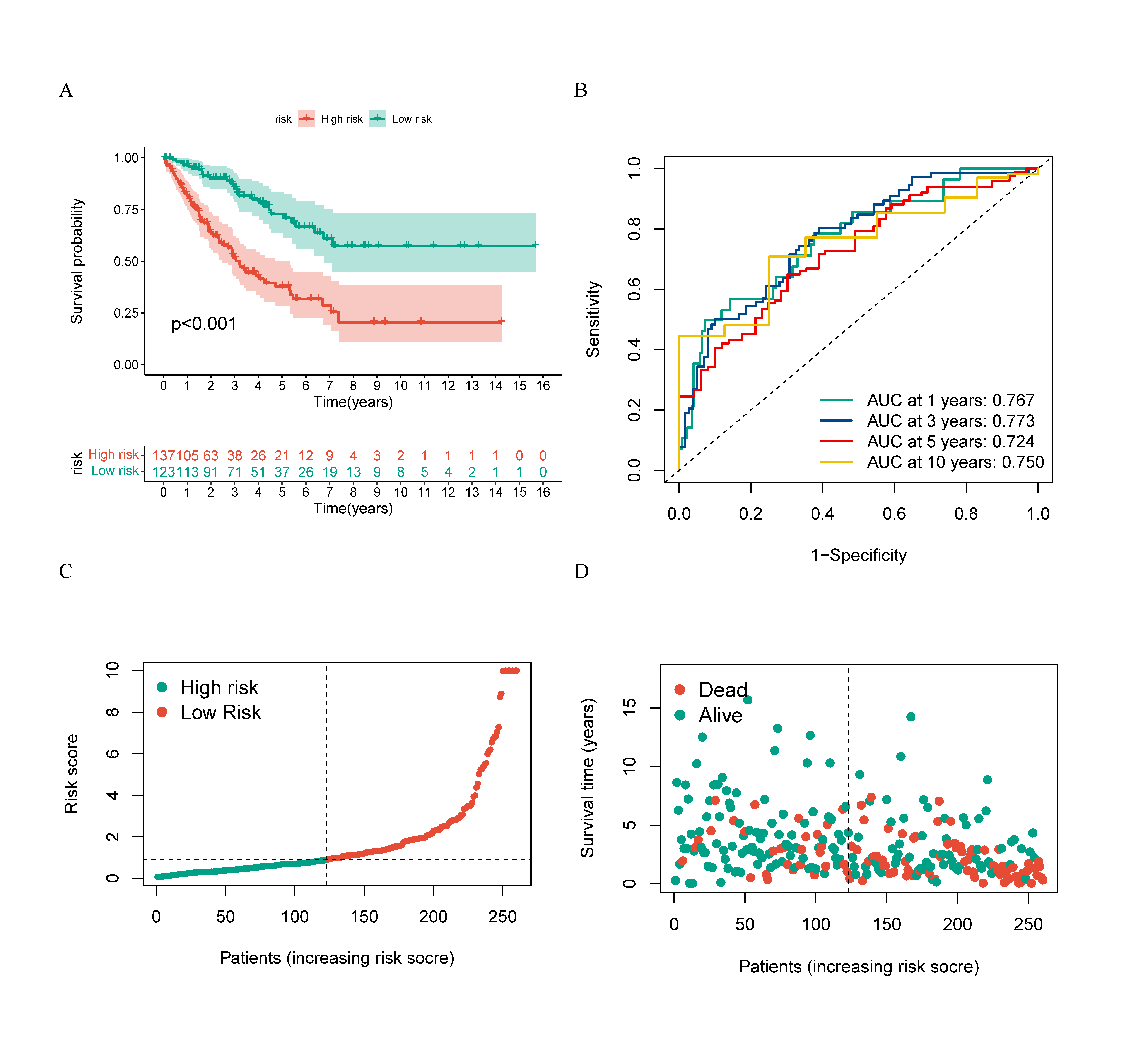

Supplement: Supplementary file 2 [file Image3.TIF]

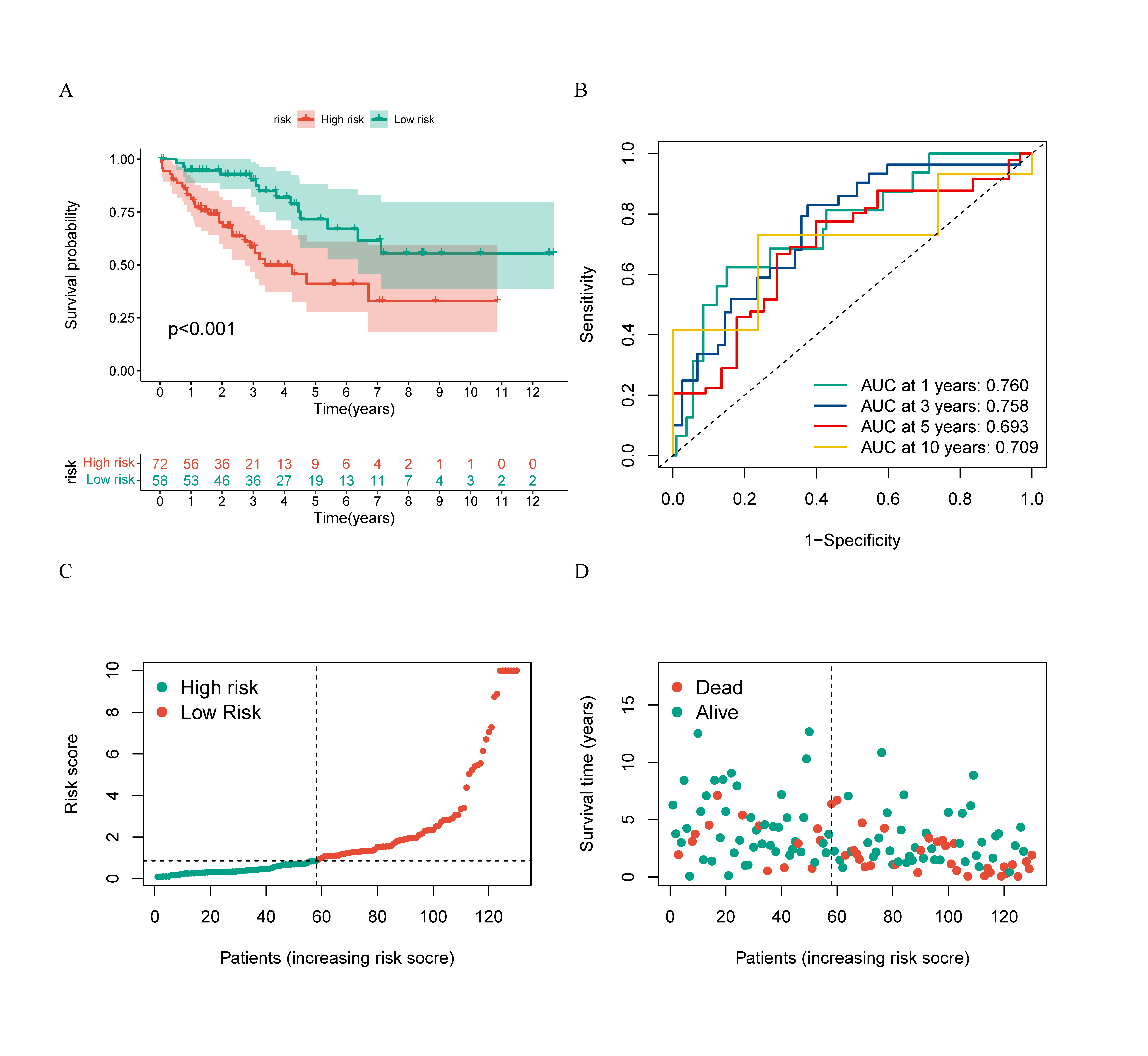

Supplement: Supplementary file 4 [file Image2.TIF]

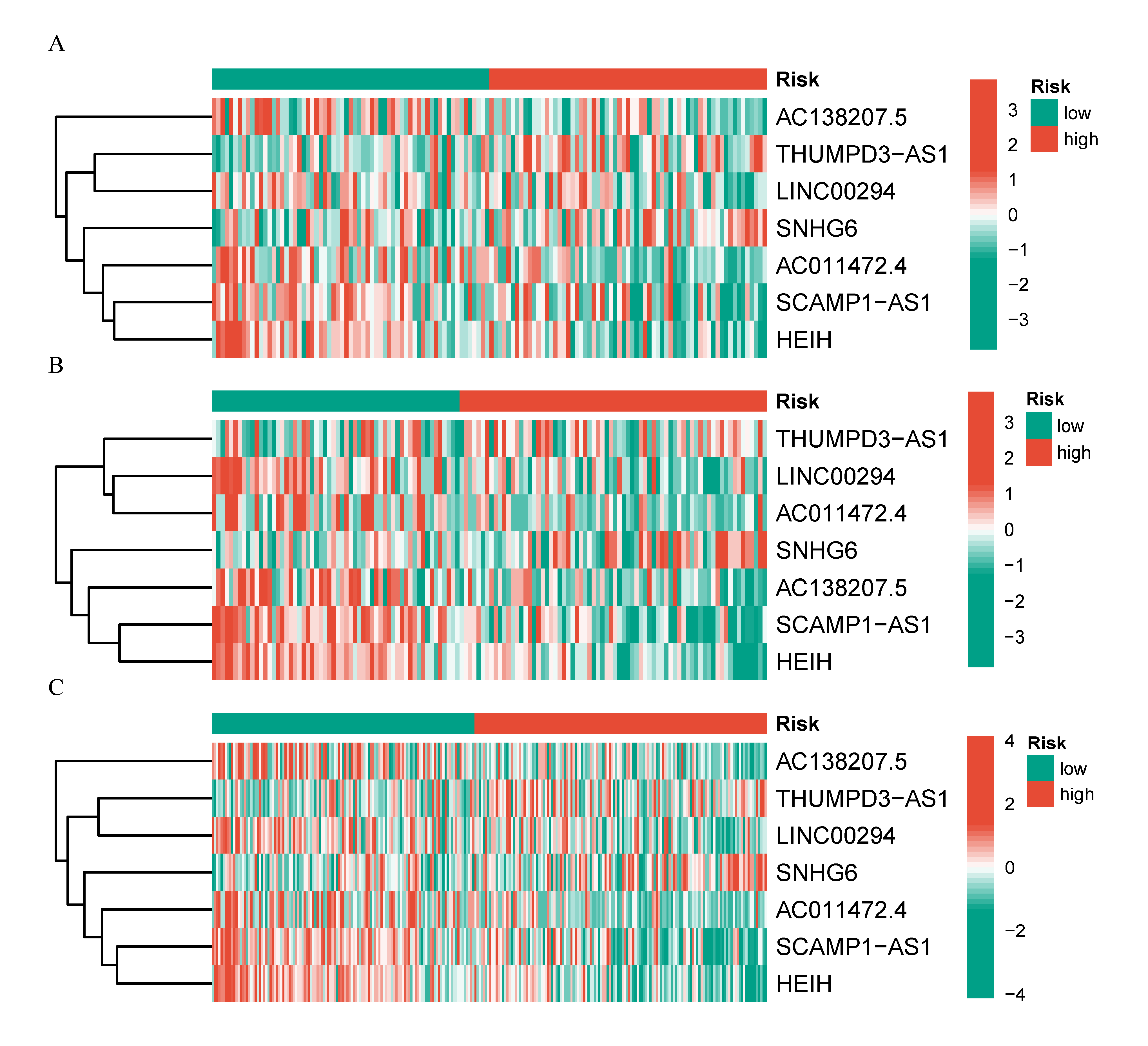

Supplement: Supplementary file 7 [file Image5.TIF]
